# Population survey sampling methods in a rural African setting: measuring mortality

**DOI:** 10.1186/1478-7954-6-2

**Published:** 2008-05-20

**Authors:** Edward Fottrell, Peter Byass

**Affiliations:** 1 Umeå International School of Public Health, Department of Public Health and Clinical Medicine, Epidemiology and Public Health Sciences, Umeå University, Umeå, Sweden; 2 Immpact, University of Aberdeen, Scotland, UK

## Abstract

**Background:**

Population-based sample surveys and sentinel surveillance methods are commonly used as substitutes for more widespread health and demographic monitoring and intervention studies in resource-poor settings. Such methods have been criticised as only being worthwhile if the results can be extrapolated to the surrounding 100-fold population. With an emphasis on measuring mortality, this study explores the extent to which choice of sampling method affects the representativeness of 1% sample data in relation to various demographic and health parameters in a rural, developing-country setting.

**Methods:**

Data from a large community based census and health survey conducted in rural Burkina Faso were used as a basis for modelling. Twenty 1% samples incorporating a range of health and demographic parameters were drawn at random from the overall dataset for each of seven different sampling procedures at two different levels of local administrative units. Each sample was compared with the overall 'gold standard' survey results, thus enabling comparisons between the different sampling procedures.

**Results:**

All sampling methods and parameters tested performed reasonably well in representing the overall population. Nevertheless, a degree of variation could be observed both between sampling approaches and between different parameters, relating to their overall distribution in the total population.

**Conclusion:**

Sample surveys are able to provide useful demographic and health profiles of local populations. However, various parameters being measured and their distribution within the sampling unit of interest may not all be best represented by a particular sampling method. It is likely therefore that compromises may have to be made in choosing a sampling strategy, with costs, logistics the intended use of the data being important considerations.

## Background

The majority of the world's people remain outside of any kind of systematic health surveillance [1,2]. In the majority of countries where the burden of disease is highest, complete surveillance remains unrealistic or unaffordable. The use of health-facility-based data as a proxy for community-based data is common but open to criticism for not being representative of the wider population in settings where the overall proportion of individuals with routine access to formal healthcare is low. The use of facility-based data can lead to large degrees of uncertainty in estimates of key parameters such as maternal mortality [3].

Population-based sample surveys and sentinel surveillance methods, such as Demographic and Health Surveys (DHS), are commonly used as substitutes for more widespread health and demographic monitoring and intervention studies [4,5]. Similarly, localised Demographic Surveillance Sites (DSS) are increasingly being acknowledged as useful surrogates for more widespread surveillance, as reflected in the growing number of DSSs constituting the Indepth Network, which has risen from 17 sites in 13 different countries at its creation in 1998 to a total of 38 separate DSSs in 19 different countries in 2007 [6-8]. Nevertheless, active follow-up of this type has been criticised for being expensive and time consuming unless it can meaningfully be extrapolated into the surrounding 100-fold population [9]. Furthermore, there remain no 'best practice' guidelines as to which survey sampling methods give the most representative samples in relation to various demographic and health parameters in rural, developing-country settings in general, and in DSSs in particular. Whilst greater understanding of the implications of sampling methods may be relevant to all survey methods, such information could be particularly useful in informing the choice of sampling strategy used in establishing DSSs and enhance the evidence-based methodology of demographic and health sample surveys conducted within established sites.

Conceptually similar to other population-based surveys, DSSs are concerned with longitudinally tracking the demographic and health indicators of individuals in a clearly defined study area through regular household surveys. Some DSSs are set up around specific intervention studies, thus the selection of the demographic surveillance area (DSA) will have already been determined. There are also examples of DSSs being established for demographic and health surveillance as the primary purpose, with the selection of the DSA being determined by logistical factors, such as distance from managing and academic institutions, as well as scientific factors, such as trying to select a DSA that may reflect wider local or national diversity and population distributions. Once the DSA has been selected, the way in which populations are sampled within study areas varies greatly between sites. For example, the Butajira Rural Health Programme (BRHP) DSS in Ethiopia is based on 10 communities within the entire Butajira DSA. This sample of communities covers approximately 10% of Butajira district and is relatively dispersed geographically, with the selected communities ranging from lowland to highland and rural to semi-urban [7,10]. In contrast, several DSSs within Indepth are more contiguous, with entire populations within the selected DSA being surveyed. For example, the Agincourt DSS in South Africa covers all villages, households and individuals with in the Agincourt sub-district [11].

Gathering valid and representative data on mortality and its risk factors through DSSs and other population sample-based surveys is key to epidemiology and to the planning, implementation and evaluation of health programmes in otherwise data-poor settings. Nevertheless, the specific reasons for collecting mortality data and end-user needs vary considerably [12]. Similarly, a wide variety of sampling procedures exist, not least with regards to their complexity [13-15]. It does not necessarily follow, however, that sampling methods are selected to suit the ultimate aims of the survey, and more complex methods may often be subjectively perceived as being 'better'. This can result in additional costs and delays in the survey, especially in resource-poor settings where the necessary expertise may be lacking. If sampling methods are unlikely to have any substantial impact on the interpretation of the data and conclusions drawn from them then simpler sample survey methods may in fact be better in terms of accessibility and adequacy for purpose.

Empirical modelling of population sampling using the English national census highlighted the potential effects of various sampling methods and demonstrated that it is possible to achieve representative data by taking 1% of a national population in a sentinel surveillance approach [16]. However, England is very different in many respects from countries that might wish to implement sentinel or DSS strategies and the effects of sampling methods that are specific to rural, developing-country settings warrant further investigation. Therefore, building on previous work and with a particular focus on measuring mortality, this paper explores the effects of different sampling procedures on the representativeness of 1% population samples in rural Africa.

## Methods

Formal statistical methods can only be used as a theoretical framework for designing survey samples where there is adequate prior knowledge at the population level. Therefore, this study applied an empirical approach to the evaluation of various survey sampling methods, using data from a large household census carried out in Burkina Faso in 2006 as part of a wider safe-motherhood evaluation study conducted by Immpact [17] and described in detail elsewhere [18]. The census, which aimed to cover the entire population in two provinces in south-eastern Burkina Faso (Koupélogo and Tapoa), registered a total of 86,378 households and 512,298 individuals, giving an average of 6.0 persons per household.

Ouargaye town, the provincial centre of Koupélogo, is approximately 230 km from the national capital, Ouagadougou, and the province borders Togo to the south. Diapaga town, the provincial centre of Tapoa, is approximately 370 km from Ouagadougou, and the province borders Benin to the south and Niger to the east. The two areas are very similar in terms of social systems, infrastructure and physical geography, with many features common to rural settings across the African continent [18,19]. As is typical in Burkina Faso, the two provinces are divided into three main administrative levels: 16 '*départements*' (8 in each province), which can be considered as districts; 507 '*zones dénombrement' *(ZDs) which are enumeration areas roughly equating to villages; and 44,072 '*concessions*', which are clusters of individual households ('*ménages*') within a ZD.

From the large number of parameters captured in the census, a selection was made in an attempt to represent the range of different variables and their associated distributions that are of key importance to demographic and health surveys, with a particular emphasis on parameters relevant to understanding mortality patterns and risk factors. The selected parameters were gender (proportion of males), age (proportion under 5 years), education (proportion of population who have completed secondary level education or above), economics (proportion in the lowest wealth quintile) and number of maternal deaths that occurred in the last 5 years, which were identified using a verbal autopsy (VA) method and computerised VA interpretation method [20]. In addition, the age- and sex-specific parameter of number of adult female residents was selected, as this is essential in measuring risk factors associated with reproductive and maternal health and enables the calculation of maternal mortality rate (MMR) [21].

A range of commonly used survey sampling methods exist, seven of which have been used in this study. The simplest method is to make a random selection of administrative units until the target population is reached. A more complex procedure of sampling with probability proportional to size (PPS) increases the probability of sampling more populous units, in an attempt to make any individual's chance of being included in the sample similar, irrespective of the size of the unit in which they live[15,22]. Stratified sampling is used to ensure the fair representation of major groupings within an overall population, for example, urban and rural areas. If approximately 10% of the total population live in urban areas, for example, it may be desirable to ensure that approximately 10% of the sample drawn from the total population will come from urban areas through proportional stratified sampling. In an attempt to emulate a typical DSS situation, where a sample tends to be drawn at a local rather than national level, a model of multi-stage 'DSS sampling' has been applied in which sampling units were drawn both randomly and using PPS from a randomly selected département. Finally, a geographically dispersed sampling method which models multi-centre studies was applied, also on a multistage basis whereby two départements were selected at random and approximately half of the target population was sampled from each, using either simple random and PPS methods. Table 1 outlines the sampling techniques employed and how they relate to real life field surveys. As in previous work, more sophisticated variations of these basic population-sampling methods have not been considered for modelling in this study since their application in developing countries has been limited.

**Table 1:** Summary of sampling methods used, outline of sampling technique and example of how the sampling technique relates to real field survey and DSS designs.

Sampling Method	Technique	Example situation in field surveys
Simple Random	Step 1: Assign a random number to each sampling unit	Cross-sectional surveys within DSS settings
	Step 2: Sort sampling units by their random number	
	Step 3: Select sampling units in ascending order of random numbers until desired sample size is reached	
Probability Proportional to Size	Step 1: Assign a random number to each sampling unit	
	Step 2: Multiply the population of each sampling unit by the random number	
	Step 3: Sort sampling units on the number generated in Step 2	
	Step 4: Select sampling units in descending order of number generated in Step 2 until desired sample size is reached	
Proportional Stratified Sampling	Step 1: Determine the proportion of sampling units needed in each strata	Cross-sectional surveys within DSS settings or establishing a DSS
	Step 2: Assign a random number to each sampling unit	
	Step 3: Select sampling units from each strata using simple random methods until the desired sample size and ratio between strata is obtained	
Multi-stage Sampling (Stage 1 random; Stage 2 random)	Step 1: Randomly select geographical area for sampling	Establishing a DSS
	Step 2: Assign a random number to each sampling unit in the selected area	
	Step 3: Sort sampling units by their random number	
	Step 4: Select sampling units in ascending order of random number until desired sample size is reached	
Multi-stage Sampling (Stage 1 random; Stage 2 PPS)	Step 1: Randomly select geographical area	
	Step 2: Assign a random number to each sampling unit in the selected area	
	Step 3: Multiply the population of each sampling unit by the random number	
	Step 4: Sort sampling units on the number generated in Step 3	
	Step 5: Select sampling units in descending order of number generated in Step 3 until desired sample size is reached	
Geographically Dispersed (Stage 1 random; Stage 2 random; Stage 3 random)	Step 1: Randomly select two geographical areas	Multi-centre study
	Step 2: Assign a random number to each sampling unit in each of the selected areas	
	Step 3: Sort sampling units by their random number	
	Step 4: Select sampling units in ascending order of random number until 50% of the desired sample is selected from each geographical area	
Geographically Dispersed (Stage 1 random; Stage 2 random; Stage 3 PPS)	Step 1: Randomly select two geographical areas	
	Step 2: Assign a random number to each sampling unit in each of the selected areas	
	Step 3: Multiply the population of each sampling unit by the random number	
	Step 4: Sort sampling units on the number generated in Step 3	
	Step 5: Select sampling units in descending order of number generated in Step 3 until 50% of the sample is selected from each geographical area	

Modelling of these sampling strategies using the Burkina Faso data was carried out by drawing 20 repeated random samples according to the above strategies, using either ZD or concession as the sampling unit and stratifying between the relatively 'urban' areas of Ouargaye and Diapaga towns and the remaining départements. Each of the total 280 samples was then analysed by the individual parameters, and the results for each sampling approach were compared with the 'gold standard' of the complete census. The concept of accuracy within the samples, i.e. the extent to which a particular sample represents the whole population, was evaluated according to whether the mean of the 20 samples from each sampling approach lay within a particular tolerance of the unsampled value.

Data were extracted from the Immpact database and only cases with complete information on each of the variables of interest were used for modelling, giving a total population of 85,428 households and 512,878 individuals. Data were aggregated at concession, ZD and département level and repeated 1% random samples were drawn as above using SPSS version 13 syntax routines. On the basis of the impracticability of surveying part units, the concept of 1% sampling was taken to mean the selection of whole sampling units until the total sampled population exceeded the 1% target.

## Results

**Figure 1:**
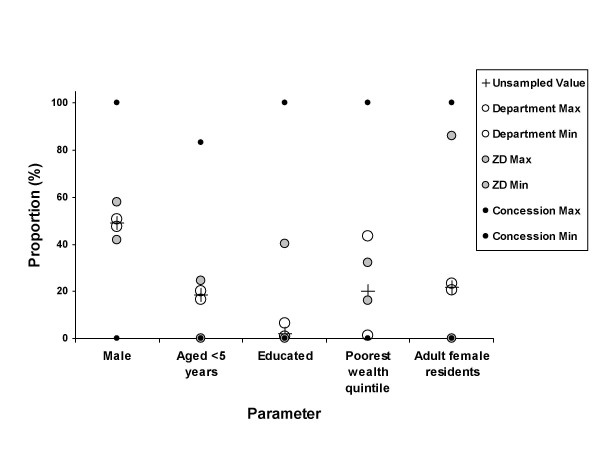
Overall proportions and maximum and minimum values of selected parameters disaggregated by three administrative levels.

Table 2 shows the mean, maximum and minimum values and their proportions for each of the key parameters by each level of disaggregation, as well as the overall population values. Figure 1 gives an indication of the distribution of these parameters, with minimum and maximum values within each level of disaggregation.

**Table 2:** Selected parameters from the survey disaggregated by three administrative levels: département, ZD and concession.

Parameter	Overall		By Département (8 in each district)			By ZD			By Concession		
			(n = 16)			(n = 507)			(n = 44072)		
	Number	%	Mean	Max.	Min.	Mean	Max.	Min.	Mean	Max.	Min.

Population	512878	100	32054.88	52207	8785	1011.59	3498	144	11.64	185	1
Male	252624	49.26	49.21%	50.74%	47.55%	49.29%	58.03%	41.98%	50.39%	100%	0.00%
Age < 5 years	94828	18.49	18.30%	20.02%	16.54%	18.40%	24.35%	0.20%	17.15%	83.33%	0.00%
Completed secondary education or above	10352	2.02	2.33%	6.39%	0.80%	2.14%	40.14%	0.00%	3.68%	100%	0.00%
***Households***	85428	100	5339.25	8397	1520	168.50	543	22	1.94	29	1
Lowest wealth quintile	17142	20.07	18.38%	43.30%	1.03%	21.00%	86.11%	0.00%	20.65%	100%	0.00%
Adult female residents	112000	21.84	22.02%	23.40%	20.71%	21.86%	32.27%	15.90%	21.49%	100%	0.00%
Maternal Deaths (n)	488	100	30.50	63	12	0.96	8	0	0.01	2	0

Crude Maternal Mortality Rate (per 100000 adult female population)	435.71		435.71			434.57			393.70		

**Figure 2:**
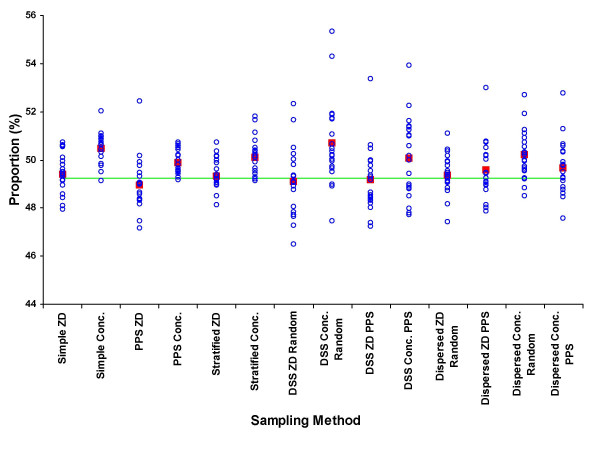
Proportion of male residents (%) by sample (blue circle), mean of 20 samples (red square), and unsampled population value (green line) for each of 7 sampling methods at two administrative levels, ZD and concession.

**Figure 3:**
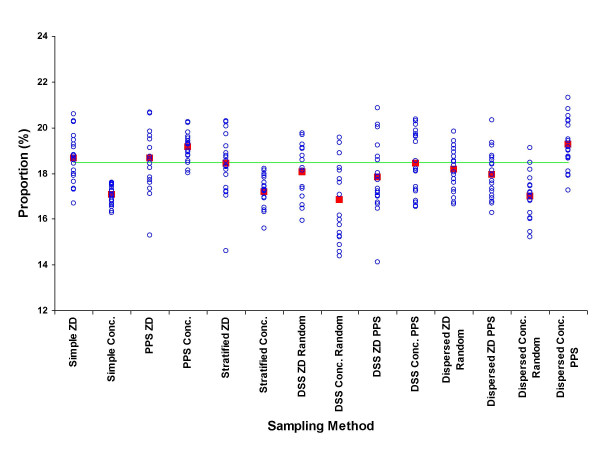
Proportion of children under 5 years of age (%) by sample (blue circle), mean of 20 samples (red square), and unsampled population value (green line) for each of 7 sampling methods at two administrative levels, ZD and concession.

**Figure 4:**
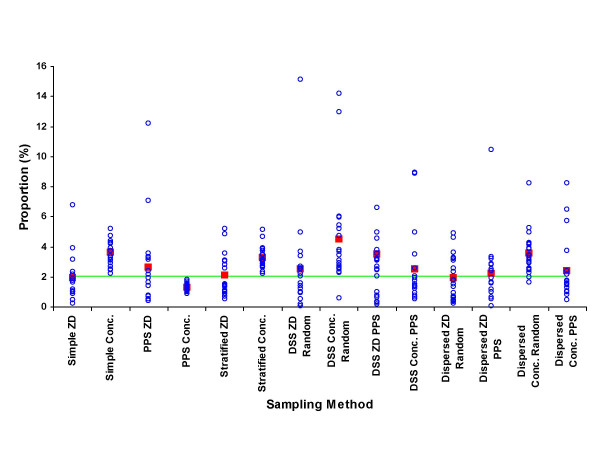
Proportion of individuals educated to secondary level or higher (%) by sample (blue circle), mean of 20 samples (red square), and unsampled population value (green line) for each of 7 sampling methods at two administrative levels, ZD and concession.

**Figure 5:**
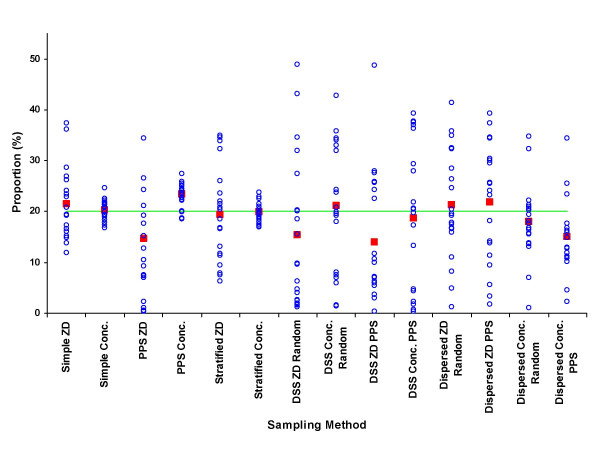
Proportion of households in the poorest wealth quintile (%) by sample (blue circle), mean of 20 samples (red square), and unsampled population value (green line) for each of 7 sampling methods at two administrative levels, ZD and concession.

**Figure 6:**
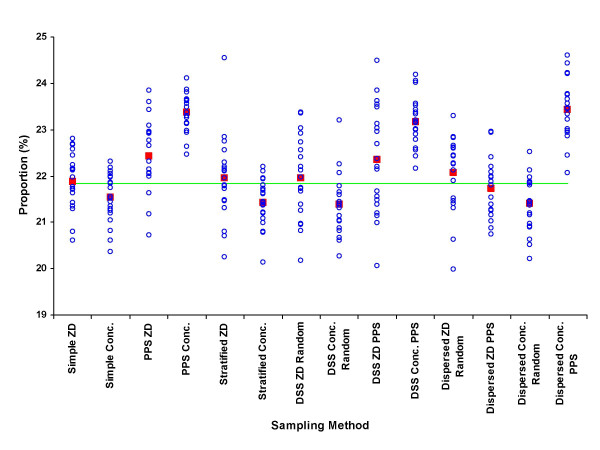
Proportion of adult female residents (%) by sample (blue circle), mean of 20 samples (red square), and unsampled population value (green line) for each of 7 sampling methods at two administrative levels, ZD and concession.

**Figure 7:**
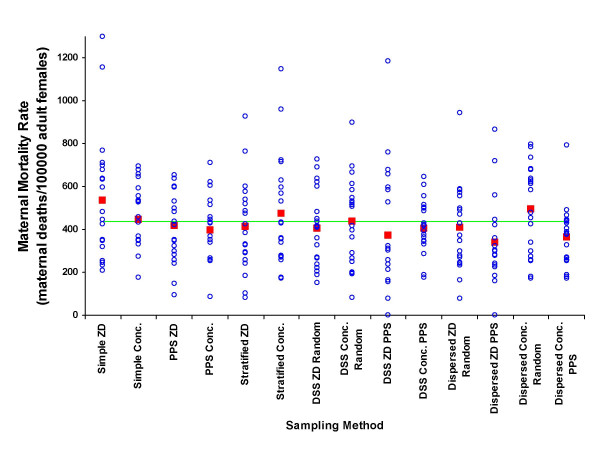
Maternal mortality rate by sample (blue circle), mean of 20 samples (red square), and unsampled population value (green line) for each of 7 sampling methods at two administrative levels, ZD and concession.

The results from the means of the 280 samples are shown as percentages of the true unsampled value for each parameter by each sampling approach in Table 3. The detailed distributions of samples by each approach for the percentage of males, under-fives, educated, wealth-quintile, adult female residents and maternal mortality rate are shown in figures 2 to 7, respectively.

**Table 3:** Mean results of 280 samples as a percentage of the unsampled value for each of the key parameters.

Parameter		Simple Random		PPS		Stratified		DSS Multistage				Dispersed Multistage			
	
		ZD	Conc.	ZD	Conc.	ZD	Conc.	ZD Random	ZD PPS	Conc. Random	Conc. PPS	ZD Random	ZD PPS	Conc. Random	Conc. PPS
	Mean number of units	5.65	440.95	2.25	66.85	5.70	437.20	5.55	4.10	458.50	174.80	5.75	3.35	464.20	157.70
	Mean Sample Population (%)	951.50 (1.11)	855.15 (1.00)	996.60 (1.13)	858.85 (1.01)	928.35 (1.09)	855.65 (1.00)	971.35 (1.14)	941.85 (1.10)	855.65 (1.00)	856.35 (1.00)	917.50 (1.07)	970.25 (1.14)	854.10 (1.00)	854.85 (1.00)
Male	Mean%	100.26	102.48	99.39	101.26	100.16	101.75	99.70	99.80	102.94	101.66	100.20	100.67	101.95	100.77
Aged < 5 years	Mean%	100.87	92.32	100.97	103.68	99.73	92.97	97.73	96.43	91.18	99.78	98.27	97.08	91.94	104.22
Educated to secondary level or higher	Mean%	94.06	179.70	130.20	62.87	104.95	160.89	124.26	172.28	222.77	123.27	95.05	109.90	176.73	118.81
Lowest Wealth Quintile	Mean%	107.22	100.95	73.49	116.94	96.96	99.20	76.23	69.96	105.03	92.97	105.93	108.37	89.94	75.24
Adult female residents	Mean%	100.18	98.58	102.66	107.01	100.50	98.08	100.50	102.34	97.94	106.04	101.01	99.45	98.03	107.28
Maternal Deaths	Mean%	105.69	203.05	101.50	127.23	104.57	203.05	104.20	103.17	203.05	159.09	104.53	103.19	203.05	162.54
Crude MMR	Mean%	113.90	294.92	108.06	189.72	115.36	288.91	110.11	104.98	293.46	216.50	113.20	106.17	305.95	209.36

Table 4 shows the number of samples giving means within both 5 and 10% tolerances of the unsampled values, for each parameter and sampling approach.

**Table 4:** Accuracy of the 20 1% for six parameters samples by each of 14 sampling methods, to within 5 and 10% tolerances of the unsampled value.

Parameter (mean)	Criterion	Overall (%)	Simple Random		PPS		Stratified		DSS Multistage				Dispersed Multistage			
			
			ZD	Conc.	ZD	Conc.	ZD	Conc.	ZD Random	Conc. Random	ZD PPS	Conc. PPS	ZD Random	ZD PPS	Conc. Random	Conc. PPS
Male (49.25%)	Samples ± 5% (%)	264 (94.29)	20 (100)	19 (95)	19 (95)	20 (100)	20 (100)	19 (95)	18 (90)	16 (80)	19 (95)	18 (90)	20 (100)	19 (95)	18 (90)	19 (95)
	Samples ± 10%	278 (99.29)	20 (100)	20 (100)	20 (100)	20 (100)	20 (100)	20 (100)	20 (100)	18 (90)	20 (100)	20 (100)	20 (100)	20 (100)	20 (100)	20 (100)
	(%)
Aged < 5 years (18.49%)	Samples ± 5% (%)	120 (42.86)	12 (60)	1 (5)	10 (50)	12 (60)	10 (50)	6 (30)	10 (50)	8 (40)	7 (35)	6 (30)	13 (65)	11 (55)	4 (20)	10 (50)
	Samples ± 10%	233 (83.21)	19 (95)	15 (75)	15 (75)	20 (100)	19 (95)	15 (75)	17 (85)	11 (55)	17 (85)	17 (85)	20 (100)	18 (90)	14 (70)	16 (80)
	(%)
Educated to secondary level or higher (2.02%)	Samples ± 5% (%)	8 (2.86)	0	0	1 (5)	0	0	0	2 (10)	0	1 (5)	2 (10)	0	1 (5)	1 (5)	0
	Samples ± 10%	20 (7.14)	2 (10)	1 (5)	4 (20)	0	0	1 (5)	3 (15)	0	1 (5)	3 (15)	1 (5)	1 (5)	1 (5)	2 (10)
	(%)
Lowest Wealth Quintile (20.07)	Samples ± 5% (%)	43 (15.36)	3 (15)	7 (35)	2 (10)	3 (15)	2 (10)	9 (45)	2 (10)	4 (20)	0	3 (15)	3 (15)	0	5 (25)	0
	Samples ± 10%	64 (22.86)	3 (15)	14 (70)	3 (15)	5 (25)	5 (25)	12 (60)	3 (15)	4 (20)	0	4 (20)	3 (15)	1 (5)	7 (35)	0
	(%)
Adult female residents (21.84%)	Samples ± 5% (%)	194 (69.29)	19 (95)	18 (90)	13 (65)	2 (10)	17 (85)	19 (95)	16 (80)	16 (80)	11 (55)	7 (35)	17 (85)	18 (90)	17 (85)	4 (20)
	Samples ± 10%	271 (96.79)	20 (100)	20 (100)	20 (100)	19 (95)	19 (95)	20 (100)	20 (100)	20 (100)	19 (95)	18 (90)	20 (100)	20 (100)	20 (100)	16 (80)
	(%)
Maternal Mortality Rate (per 100000 adult female residents) (435.68)	Samples ± 5% (%)	32 (11.43)	2 (10)	2 (10)	2 (10)	6 (30)	2 (10)	2 (10)	3 (15)	1 (5)	0	3 (15)	1 (5)	2 (10)	2 (10)	4 (20)
	Samples ± 10%	41 (14.64)	2 (10)	3 (15)	2 (10)	6 (30)	3 (15)	2 (10)	5 (25)	2 (10)	0	4 (20)	2 (10)	2 (10)	2 (10)	6 (30)
	(%)
Overall (%)	Samples ± 5%		56 (46.67)	47 (39.17)	47 (39.17)	43 (35.83)	51 (42.50)	55 (45.83)	51 (42.50)	45 (37.50)	38 (31.67)	39 (32.50)	54 (45)	51 (42.50)	47 (39.17)	37 (30.83)
	(%)	
	Samples ± 10%		66 (55)	64 (53.33)	70 (58.33)	66 (55)	70 (58.33)	68 (56.67)	55 (45.83)	57 (47.50)	66 (55)	66 (55)	62 (51.67)	64 (53.33)	60 (50)	73 (60.83)
	(%)	

## Discussion

By empirically modelling survey sampling procedures on a large dataset from a rural African setting this study has attempted to evaluate several commonly used sampling methods with regard to how well samples represent the 'true' unsampled population value of various parameters. Overall, all sampling methods and parameters tested here performed reasonably well in representing the overall population. Nevertheless, a degree of variation could be observed both between sampling approaches and between different parameters.

As demonstrated in a similar study using English census data [16] the reliability of samples between parameters was related to the overall distribution of the parameters in the dataset. The consistent and approximately normal distribution of gender meant that the proportion of males in the population was well represented in the samples, irrespective of the sampling approach (figure 2). In contrast, the more skewed and inconsistent distribution of educated individuals resulted in few samples adequately reflecting the overall situation in terms of falling within 5 and 10% tolerance of the unsampled value (table 4).

The strategies presented in this study relate to the level above the household level and data from all households within selected concessions or ZDs were summarised once the concession or ZD had been chosen. However, the mean size of concessions is approximately 12 individuals (table 2) and, given that in many field surveys and in most DSSs, households are defined as the group of people who eat together rather than by physical house structures [23], results for concession-level sampling may be considered as household-level sampling in a rural African setting. Nevertheless, it is important to acknowledge that if one were interested in individual-level parameters, such as individual risks, the sampling strategies might produce different results. It is also important to emphasise that this study did not attempt to address the issue of sample size, rather the 1% sample size used in this study relates to the premise that active follow-up in DSSs can only be justified if it can meaningfully be extrapolated into the surrounding 100-fold population [9]. It is likely that this sample size would be either too small or too large to address certain measurement needs, such as under-five mortality estimates based on a birth history.

The DSS-style sampling in this modelling was emulated using a multi-stage approach, selecting a département at random and then selecting ZDs or concessions within the département either randomly or using PPS. The first stage of the process thus established the equivalent of a locality for the DSS, which was then sampled locally. Several outliers are associated with this approach to sampling in a number of parameters where the urban département of Diapaga was randomly selected in the first stage of this two-stage method. In particular, multi-stage DSS-style methods in which individual units were selected randomly are notable for the clear outlying samples at both the ZD and concession level with regard to the educational level parameter caused by selection of Diapaga in the first stage of sampling (figure 4). These outliers overestimate the true population value and may be explained by the fact that, as the provincial capital, Diapaga is the biggest town in this area and has a concentration of secondary schools and a higher demand for an educated workforce. The simple PPS sample at the ZD level also has an obvious education outlier, which is also caused by the selection of Diapaga. In this outlying sample Diapaga comprises one third of the selected sampling units and distorts the mean estimation of the proportion of educated individuals because over 30% of the individuals within the sample from Diapaga were educated to secondary level or above. It is clear from table 4 that DSS multistage methods were the worst performing in terms of representing the unsampled population but were somewhat improved by the dispersed multistage modelling between two randomly selected départements. Perhaps more thought needs to be given to this kind of 'distributed DSS' strategy in which sampling nodes could be more widely distributed.

Stratification methods are intended to limit disproportionate selection of units from obvious strata. In the case of education, stratification between 'urban' and rural areas appeared to overcome the problem of outliers associated with several of the other methods by limiting the selection of sampling units from urban areas. Overall however, stratification did not appear to greatly influence the accuracy of samples in a positive or negative way. This may be due to the fact that stratification between urban and rural areas in this part of Africa may be somewhat artificial, with descriptions of urban areas subjectively relating to an area being 'less rural' than its surrounding areas.

Samples drawn at the ZD level were generally closer to the unsampled population values than samples drawn at the concession level for all parameters except for the proportion of households in the poorest wealth quintile, where concession level samples were more representative (figure 5). A possible explanation for this may be that economics are more homogenous within areas than between areas, thus selecting a greater number of smaller and potentially more diverse areas will produce an overall estimate more representative of the wider population. The same reason may explain the inconsistent performance of multi-stage DSS sampling in measuring wealth, which produced a wide range of estimates determined by the department within which the second stage of sampling was confined. Geographically dispersed DSS sampling improved reliability to some extent, again reflecting the need to consider general population distributions and accommodate for uniformity of certain parameters within localities.

Random methods of each of the specific sampling methods generally performed better than PPS methods, especially at the ZD level. This may be due to systematic errors in PPS methods if certain parameters are markedly different in more densely populated sampling units. Although PPS methods are theoretically appealing on the basis of providing every individual with a more equal chance of being sampled, the practical benefits of this over more simplistic methods is questionable in developing-country settings where extremes of distribution of certain parameters, such as wealth, are often more commonplace and associated with localised extremes of population density.

Interestingly, PPS approaches proved more reliable and generally more representative at the concession level than random approaches at the same administrative level with regard to the proportion of educated individuals (figure 4) and the proportion of under-fives (figure 3). This is in contrast to the pattern observed at the ZD level. With regard to the proportion of individuals educated to secondary level or above, the superior performance of PPS approaches at the concession level compared to random approaches at the same administrative level may be explained by the fact that the distribution of education is more homogenous in less populated concessions, therefore increasing the likelihood of selecting more populous units (which have a more heterogeneous education distribution) PPS methods gives more representative samples.

The mean population of concessions with at least one individual aged less than 5 years is significantly higher than those concessions with no under-fives (13.23 vs. 5.20, p < 0.0001). This is perhaps not surprising since such concessions are more likely to be comprised of family units and must always include the under-fives plus at least one carer. Random approaches to each of the sampling methods at concession level consistently gave a mean of the samples that underestimated the proportion of under-fives (figure 3). PPS methods at the same administrative level performed better by increasing the likelihood of selection more populous units (where more under-fives live).

Education has well-established associations with mortality and under-five mortality is a common health and development index. As such, unrepresentative measurements of these parameters could have important implications on reliable mortality measurements. Therefore this study suggests that sample surveys aiming to measure skewed parameters or parameters intuitively more common in more populous areas should give careful consideration to the benefits of PPS methods. Further investigation with a wider range of parameters with various population distributions and from different settings is appropriate.

In measuring the proportion of adult female residents, sampling at the ZD level was consistently better than sampling at the concession level and PPS methods produced estimates further from the true population mean (figure 6). However, since a greater number of maternal deaths will occur in a population with a larger population at risk (i.e. adult female residents), selecting a sample that misrepresents the true population of adult female residents is also likely to misrepresent the number of maternal deaths in the same population, thus the overall effect on actual MMR estimates may be largely self adjusting. This appears to be the case since none of the mean results of each sampling method gave particularly unsatisfactory results for maternal mortality rate estimates (figure 7). Maternal health measurements with the purpose of assessing risk factors, causal pathways and designing interventions, however, are concerned with more than simply determining MMR. Population level risk factors associated with the every day lives of women are likely to be misrepresented if the proportion of adult females itself is misrepresented. For these reasons the results from this modelling suggest that sampling a greater number of smaller units (concessions in this case) using PPS methods may not be the most appropriate method for maternal health studies at the community level.

In addition to the variation in reliability and representativeness identified in this study, the effects of different sampling methods should also be discussed in terms of the intended use of data from health and demographic sample surveys in resource-poor settings. If the purpose of such surveys is to gain an overall impression of population composition and distribution of risk factors to inform public-health policy and intervention planning in a simple and cost-effective way, then misrepresentation of the population may only be important if the conclusions one would draw from the results would be effected [24]. In this respect it is difficult to envisage that any of the samples drawn in this study would have greatly changed the conclusions drawn about age and sex distribution, wealth and education, and maternal mortality in this setting. This is important and raises the question of whether more complex methods are worth the extra effort and expertise that they demand.

Cost and logistical considerations are also important. Multi-stage sampling methods, for example, are cheaper and are often the only realistic option for undertaking research in rural African settings, even at the expense of statistical precision as suggested by this study. In public health terms, dispersed methods such as simple random sampling would not only be impractical in terms of intervention measures but could also diminish the social force that a more unified study population might use as a lever for action.

Strict epidemiological data analysis should reflect the sampling strategy employed and it may be interesting to investigate the effects of different sampling strategies on more complex statistical analyses, such as multivariate and multilevel modelling. The practical importance of not taking design effects into consideration when analysing data and how this may influence the usefulness of the data for different end-user perspectives remain important questions, the answers to which may be of great relevance to field research in countries lacking sampling frames. Sampling design and choice of appropriate designs for population surveys in rural African settings could be improved by a better understanding of basic population parameter distributions through empirical studies of these issues using practical (preliminary surveys; use of census data) as well as theoretical (population modelling) techniques. More extensive modelling using large existing data sets may create opportunities for generating realistic population simulations that could enable more sophisticated understanding of regular biases associated with differing methods, and subsequently more evidence-based selection of sampling strategies.

## Conclusion

Sample surveys are able to provide useful demographic and health profiles of local populations and, to be cost-effective, need be generalisable to the surrounding population. Sampling strategies are thus an important consideration, but various parameters being measured and their distribution within the sampling unit of interest may not all be best represented by a particular sampling method. It is likely therefore that compromises may have to be made in choosing a sampling strategy. Simple sampling approaches are not always less appropriate than more complex methods and are able to provide useful information for local public health planning, monitoring and evaluation, whilst needing less specialist expertise. Understanding the potential advantages and limitations of possible sampling methods in particular contexts is important for avoiding inappropriate population survey designs, particularly in settings lacking sampling frames.

## Abbreviations

DHS: Demographic and Health Survey; DSS: Demographic Surveillance Site; DSA: Demographic Surveillance Are; ZD: Zone dénombrement; VA: Verbal Autopsy; MMR: Maternal Mortality Rate; PPS: Probability Proportional to Size (sampling method); Conc. (in figures only): Concession.

## Competing interests

The authors declare that they have no competing interests.

## Authors' contributions

Both authors were involved in data acquisition and design of the study. EF performed data manipulation, analysis, and interpretation and drafted the first version of the manuscript. PB contributed significantly to the interpretation of findings and revision of the manuscript. Both authors read and approved the final manuscript.
